# Species level composition of *Faecalibacterium* spp. in the gut of Japanese adults revealed by *rpoA*-based sequencing analysis

**DOI:** 10.1093/femsec/fiag049

**Published:** 2026-05-09

**Authors:** Adeline Ang, Eri Mitsuyama, Hiroki Kaneko, Toshitaka Odamaki, Akihito Endo

**Affiliations:** Department of Nutritional Science and Food Safety, Graduate School of Applied Bioscience, Tokyo University of Agriculture, Tokyo 156-8502, Japan; Biotics Research Institute, Research & Development Division, Morinaga Milk Industry Co Ltd., Kanagawa 252-8583, Japan; Biotics Research Institute, Research & Development Division, Morinaga Milk Industry Co Ltd., Kanagawa 252-8583, Japan; Biotics Research Institute, Research & Development Division, Morinaga Milk Industry Co Ltd., Kanagawa 252-8583, Japan; Department of Nutritional Science and Food Safety, Graduate School of Applied Bioscience, Tokyo University of Agriculture, Tokyo 156-8502, Japan

**Keywords:** *Faecalibacterium* spp, *Faecalibacterium prausnitzii*, *rpoA* sequencing, species-level composition, BMI, diet intake

## Abstract

*Faecalibacterium* is a predominant genus in the human gut that potentially supports intestinal and systemic health. Recent genomic studies revealed heterogeneity within the genus, leading to the recognition of additional species beyond the type species, *Faecalibacterium prausnitzii*. Despite its biological importance, the species-level distribution and ecological diversity of *Faecalibacterium* spp. remains poorly characterized, partly due to methodological constraints. Here, we developed a *rpoA*-based primer set specific to the *Faecalibacterium* genus and applied on fecal samples from 580 healthy Japanese adults for species-level profiling with next-generation sequencing. Our results revealed clear distinctions in species-level population, as well as inter-individual variations influenced by host characteristics and dietary factors. The majority of *Faecalibacterium* composition consisted of *F. longum, F. taiwanense, F. duncaniae, F. prausnitzii*, and a yet-to-be-classified species (Group 11). Notably, *F. prausnitzii* was neither the most prevalent nor the most abundant species. Besides that, host driven differences such as body mass index revealed negative association between relative abundance of *F. duncaniae* and Group 11. Additionally, Group 11 displayed lower relative abundance in lower caloric intake group. Overall, these findings highlight environment- and host-dependent shifts in *Faecalibacterium* populations, revealing species-specific ecological adaptations and potential physiological roles in human health.

## Teaser

rpoA-based sequencing revealed species-level *Faecalibacterium* composition in healthy Japanese adults.

## Introduction

*Faecalibacterium prausnitzii* is reported to be one of the most predominant butyrate producers in the healthy human gut (Miquel et al. 2013). *Faecalibacterium prausnitzii* was discovered in 2002 (Duncan et al. 2002), being described as a homogeneous species. It was proposed as a potential next-generation probiotic (Martín et al. 2017, Kumari et al. 2021) with the ability to produce butyrate and Microbial Anti-inflammatory Molecules (MAMs) (Auger et al. 2022). Butyrate functions as an energy source to colonocytes and helps support the integrity of the intestinal barrier by maintaining the tight junctions, thereby preventing pathogens and inflammatory molecules from entering the bloodstream (Singh et al. 2023). Moreover, butyrate exhibits anti-inflammatory properties by the regulating inflammatory cytokines and inducing the differentiation of T-reg cells (Ihara et al. 2017). MAMs produced by *F. prausnitzii* have been reported to modulate and suppress the NF-*κ*B pathway, thereby lowering inflammation (Auger et al. 2022). Furthermore, *F. prausnitzii* has been proposed as a potential health biomarker, with studies linking decreased abundance to the progression of diseases such as Chron’s disease (Sokol et al. 2008), ulcerative colitis (Franzosa et al. 2019), colorectal cancer (Zou et al. 2018) and atopic dermatitis (Fujimura et al. 2016).

Recent *in silico* analyses have revealed heterogeneity within the taxon, suggesting the need for reclassification of *F. prausnitzii* (Lopez-Siles et al. 2016, De Filippis et al. 2020). After the reclassification, at the time of writing (2025, May), the genus *Faecalibacterium* includes validated species such as *F. prausnitzii, F. butyricigenerans* (Zou et al. 2021)*, F. duncaniae* (Sakamoto et al. 2022)*, F. hattorii* (Sakamoto et al. 2022)*, F. langellae* (Ang et al. 2025)*, F. longum* (Zou et al. 2021)*, F. taiwanense* (Liou et al. 2024), and *F. wellingii* (Plomp and Harmsen 2024) from the human gut and *F. gallinarum* (Sakamoto et al. 2022) derived from chicken gut. The reclassification of taxon raises the question of whether the reported health benefits are specific to *F. prausnitzii* or shared by other related species. Auger et al. further reported that MAMs derived from different *Faecalibacterium* phylogroups displayed varying levels of anti-inflammatory activity (Auger et al. 2022), suggesting distinct species-dependent health effects. Therefore, additional studies are necessary to clarify the species-specific roles in host health and to identify potential health biomarker and probiotic candidates.

As part of this effort, Tanno et al. and McLellan et al. developed quantitative PCR (qPCR) primers targeting single-copy genes such as *rpoA* (Tanno et al. 2023) and *mam* (Mclellan et al. 2025), respectively, for absolute quantification of *Faecalibacterium* at the species level. The *rpoA*-based qPCR was applied in a study of Japanese adults (Hirasaki et al. 2025), whereas *mam*-based qPCR was used in a comparative analysis between Chron’s disease patients and healthy subjects (Mclellan et al. 2025). Both studies demonstrated species-level distribution and dominance within the genus, highlighting qPCR as a powerful tool for investigating *Faecalibacterium* populations. However, qPCR has limitations, including the need for prior genomic knowledge of target species when designing primers and generating standard curves. Moreover, qPCR-based studies can be time-consuming and labor-intensive, as species-specific primers and separate amplification reactions are required for each species. This makes qPCR unsuitable for large-scale cohort studies or for targeting a broad range of taxa.

Alternatively, 16S rRNA gene sequencing is widely used for analyzing the gut microbiota. However, the 16S rRNA gene sequencing faces several limitations, including insufficient resolution for species-level analysis and variable copy numbers of the gene among a single genome, which causes potential bias in quantitative analysis. Moreover, Tanno et al. reported that the 16S rRNA gene is an unsuitable gene marker for identification of *Faecalibacterium* spp. due to the high intra-strain heterogeneity and low inter-species discriminatory power, resulting in poor species-level clustering (Tanno et al. 2022). Alternatively, shotgun metagenomic sequencing provides high-resolution classification, but the high cost and computational demands often make it impractical for large-scale studies. As such, more discriminatory targeted sequencing methods are required to achieve species-level resolution in a feasible manner. The use of alternative gene markers in sequencing has proven successful to study species-level composition within specific genera and families, including the internal transcribed spacer (ITS) region for *Bifidobacterium* (Milani et al. 2014) and *gyrB* for the families *Bacteroidaceae, Bifidobacteriaceae*, and *Lachnospiraceae* (Nichols and Davenport 2024).

For these reasons, the present study developed a *rpoA*-based sequencing analysis to study composition of *Faecalibacterium* spp. We selected *rpoA*, a highly conserved gene with single copy and exhibits substantial interspecies variation which allows species-level resolution and resolves the concerns of potential quantification bias. The developed *rpoA*-based sequencing analysis was applied to explore the composition of *Faecalibacterium* spp. on a cohort of 580 Japanese adults. Additionally, we studied the relationship between species level composition of *Faecalibacterium* spp. with dietary and lifestyle habits.

## Materials and methods

### Acquisition of genomic data and primer design

To design *Faecalibacterium* genus specific primers, genomic data from two previous studies were combined. An initial total of 88 complete and draft genomes of *Faecalibacterium* strains were obtained from prior work, including 86 strains retrieved from the NCBI database (Tanno et al. 2022) and two additional strains (*F. butyricigenerans* AF52-21^T^ and *F. longum* CM04-06^T^) obtained from CNGBdb. Additionally, all complete and draft genomes of *Faecalibacterium* spp. and *F. prausnitzii* (n = 147) containing the *rpoA* gene, deposited in the NCBI database (July 2021) were further added to the original 88 strains, resulting in a total of 235 strains (Tanno et al. 2023). The 235 *Faecalibacterium* genomes were re-annotated with DFAST and *rpoA* sequences were extracted. The extracted *rpoA* sequences were aligned by using ClustalX (version 1.83), and the consensus region was targeted for primer design specific to human-origin *Faecalibacterium* spp. The final primer pair designed, meta-*rpoA*-F (5′-CACCCCBGTKCTCAAGGTGAA-3′) and meta-*rpoA*-R (5′-CCSARRTTRCGRACYTTCATC-3′), incorporated degenerate bases to improve coverage across the taxa.

### Evaluation of efficiency and sensitivity of *Faecalibacterium rpoA*-specific primers

The designed primers were first validated *in silico* using NCBI Primer-BLAST with default settings (Ye et al. 2012). Further evaluation of amplification efficiency and specificity was conducted in two steps by using qPCR. In the first step, the amplification efficiency of designed primer pairs was assessed using synthetic *rpoA* DNA. In the present study, human-origin *Faecalibacterium* spp. were categorized into 12 species-level phylogenetic groups (referred to as species). The classification consists of nine species previously proposed by Tanno *et al*. (Tanno et al. 2023), with the addition of a newly proposed novel species *F. wellingii* (Group 10) (Plomp and Harmsen 2024) and two groups (clades L and N) described by De Fillipis et al. (De Filippis et al. 2020) (Table 1). These species show average nucleotide identities below 94% among intra-species comparisons. For qPCR, synthetic *rpoA* DNA was used as a substitution to bacterial culture because specific *Faecalibacterium* species were not publicly available at the time of analysis (Oct 2024). Full-length *rpoA* sequences of 12 *Faecalibacterium* species were synthesized by insertion into pEX-A2J2 vector (Eurofins Genomic, Tokyo, Japan), as described by Tanno et al (2023). The set of 12 species included at least one representative strain from each species, with three strains included for *F. prausnitzii* (Group 1) and two strains for *F. duncaniae* (Group 6). qPCR was performed using synthesized *rpoA* DNA at a concentration of 100 pg (=7.43 log_10_  *rpoA* gene copies). FastStart Essential DNA Green MasterMix with the LightCycler96 system (Roche, Basel, Switzerland) was used, according to manufacturer’s instructions. The qPCR program consisted of an initial denaturation at 95°C for 10 min, followed by 45 cycles for 95°C for 20 s, 50°C for 10 s and 72°C for 45 s. A standard curve was generated from synthesized *rpoA* DNA of *F. prausnitzii* ATCC 27768^T^. Samples were run in triplicate on the same plate, and the mean and standard deviation (SD) were obtained.

**Table 1:** Classification of *Faecalibacterium* spp. in the present study.

Group	Species	Type strain
1	*F. prausnitzii*	ATCC 27768^T^
2	*F. langellae*	CNCM I-4541^T^
3	*F. taiwanense*	HLW 78^T^
4	*F. longum*	CM 04–06^T^
5	Not classified yet	APC942/8–14–2*
6	*F. duncaniae*	A2-165^T^
7	*F. hattorii*	APC922/41–1^T^
8	Not classified yet	MGYG-HGUT-00195*
9	*F. butyricigenerans*	AF52-21^T^
10	*F. wellingii*	HTF-F^T^
11	Not classified yet	UMGS183*
12	Not classified yet	UMGS253*

*Representative strains are indicated for the yet-to-be classified groups.

In the second step, primer pair specificity was confirmed by qPCR against DNA extracted from 11 human commensal gut bacteria and four *Faecalibacterium* species. The commensal strains included in this study were *Bifidobacterium longum* subsp. *longum* JCM 11340^T^, *Bifidobacterium adolescentis* JCM 1251^T^, *Parabacteroides distasonis* JCM 5825^T^*, Bacteroides ovatus* JCM 5824^T^, *Anaerostipes caccae* JCM 13470^T^, *Segatella copri* JCM 13464^T^, *Blautia producta* JCM 1395, *Lactobacillus gasseri* NRIC 1639^T^, *Mediterraneibacter gnavus* JCM 6515^T^, *Butyricicoccus faecihominis* JCM 31056^T^, and *Roseburia intestinalis* JCM 17583^T^. *Faecalibacterium* strains included were *F. langellae* CNCM I-4541^T^ in Group 2, *F. taiwanense* JCM 39529^T^ in Group 3, *F. longum* JCM 39211^T^ in Group 4 and *F. duncaniae* JCM 31915^T^ in Group 6. The JCM, CNCM, and NRIC strains were obtained from the Japan Collection of Microorganisms (JCM), Collection Nationale de Cultures de Microorganismes (CNCM) of Institut Pasteur, and NODAI Culture Collection Center (NRIC), respectively. Bacterial strains were cultured in GAM broth (Nissui, Tokyo, Japan) or YCFA broth at 37°C for 24 h, as described previously. The composition of YCFA broth was described elsewhere (Lopez-Siles et al. 2012). Cultured bacterial cells were harvested and broken by bead beating with a BioShaker M/BR-022 (TAITEC) in TE buffer supplemented with 0.1 mm glass beads. DNA was then extracted using phenol-chloroform and ethanol precipitation according to standard protocols. The extracted DNA was diluted to 10 ng and used for qPCR. qPCR with the meta-*rpoA* primers was conducted using the same conditions as described above.

### Study population, fecal sampling, and metadata collection

Fecal samples, human metadata and dietary intake were obtained from adult Japanese volunteers in a previous study (FAECES-02) conducted by Morinaga Milk Industry Co., Ltd. (Park et al. 2021). Written informed consent was obtained from all participants, and the study protocol was approved by the Ethics Committee of the Japan Conference of Clinical Research (Tokyo, Japan). The study was conducted in accordance with all relevant ethical guidelines.

Fecal samples were collected as previously described (Park et al. 2021) using a fecal catcher for self-collection at home without water immersion. Fecal sample of approximately a red bean-size was collected, mixed with 3 ml of guanidine thiocyanate solution (TechnoSuruga Laboratory, Shizuoka, Japan) and transported to the laboratory at room temperature. Participants also completed a questionnaire covering anthropometric data, medical history, fecal characteristics, dietary habits, and physical activity. Dietary intake was assessed by the Brief self-administered Diet History Questionnaire (BDHQ), with data calculated based on frequency over the preceding month. For this study, the “seasonal foods (KISETSU)” category was removed from the BDHQ analysis to avoid redundancy with the “fruit” category. The final analysis included 67 food and beverage items grouped into 18 different categories (Table S1).

**Figure 1:**
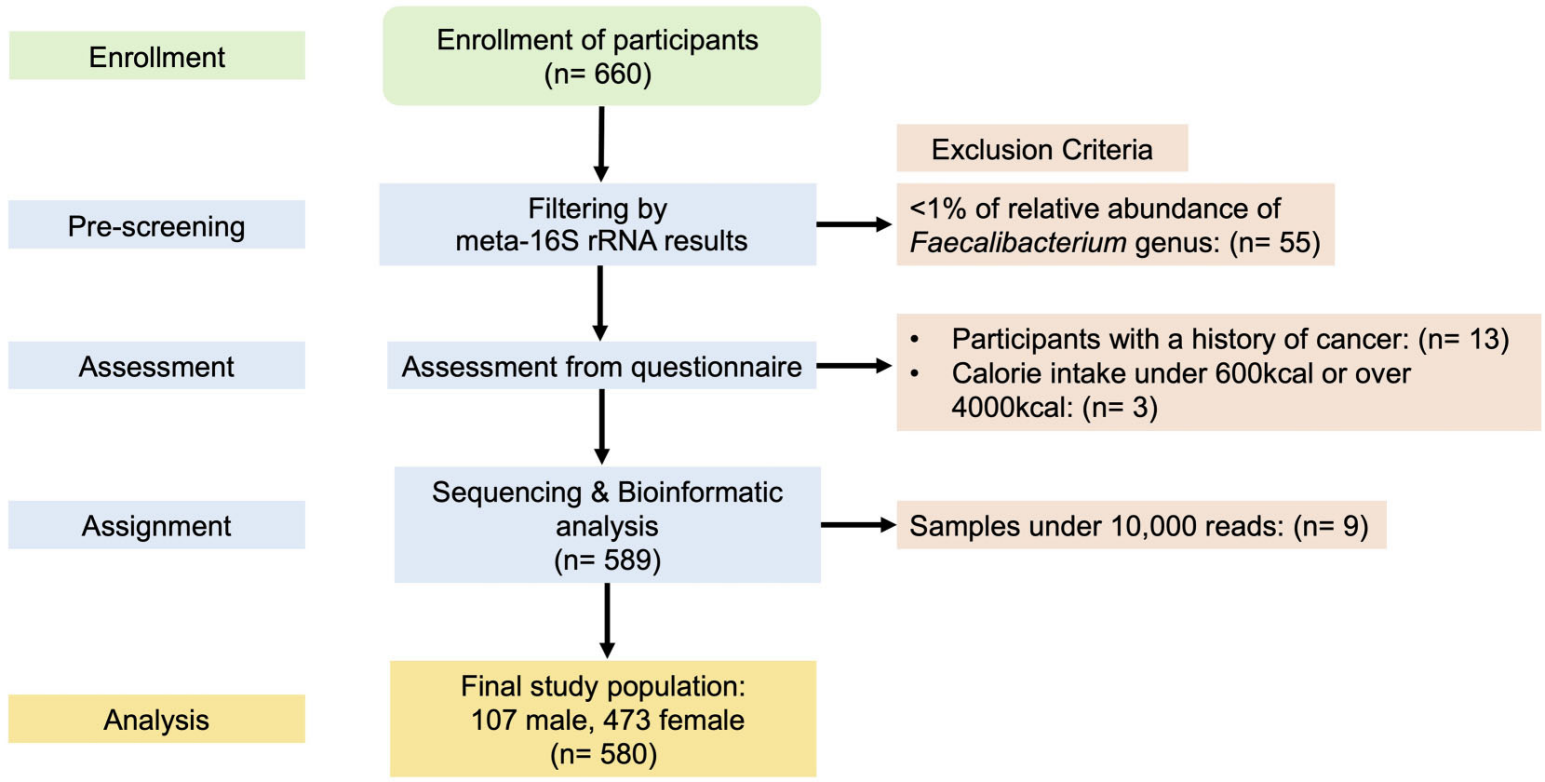
Flow chart of study protocol and sample selection.

The recruitment and screening process was summarized in Fig. 1. Participants who had taken antibiotics, laxatives, or anti-inflammatory medications within two weeks of sample collection were excluded prior to enrollment. Following the initial exclusion, 660 Japanese adults were recruited. All participants were determined to be physically independent and free of health conditions requiring medical intervention upon clinical assessment. To ensure data quality, participants with <1% relative abundance of the *Faecalibacterium* genus (based on 16S rRNA gene sequencing from Park et al. 2021) were excluded (n = 55). Further screening was conducted to exclude participants with a history of cancer (n =13). Next, dietary outliers with a total energy intake of <600 kcal or >4000 kcal were excluded (n = 3) to reduce confounding effects of extreme diets on the gut microbiome. Finally, samples with <10 000 reads were removed during bioinformatic processing (n = 9). The final study population consisted of 580 Japanese adults (107 males, 473 females), between the ages of 21 and 83.

### DNA extraction from fecal samples and *rpoA* gene sequencing

The fecal samples collected were mechanically disrupted using FastPrep-24 5 G (MP Biomedicals, Santa Ana, CA, United States), and DNA was extracted by using Gene Prep Star PI-480 device (Kurabo Industries). *rpoA* gene sequencing was performed following previously described protocols with slight modifications (Odamaki et al. 2016, Kato et al. 2018). Partial sequence of *rpoA* gene (325 bp) was amplified using the TaKaRa Ex Taq HS Kit (Takara Bio, Shiga, Japan) with meta-*rpoA*-F and meta-*rpoA*-R primer pair. The final designed primer pair included Illumina adapter overhangs were Fwd 5′-CGCTCTTCCGATCTCTGCACCCCBGTKCTCAAGGTGAA-3′ and Rev 5′-CGCTCTTCCGATCTGACCCSARRTTRCGRACYTTCATC-3′ where adapter sequences were underlined. PCR was conducted in triplicate using the following conditions: preheating at 94°C for 3 min; 30 cycles of 94°C for 30 s, 50°C for 30 s, and 72°C for 5 min. PCR products were verified by size using the QIAxcel system (Qiagen, Valencia, CA, USA). Triplicate samples were pooled together, and the combined PCR products were barcoded in the second PCR using barcoded primers adapted for Illumina-NextSeq: Fwd 5′- AATGATACGGCGACCACCGAGATCTACACNNNNNNNNACACTCTTTCCCTACACGACGCTCTTCCGATCTCTG and Rev 5′- CAAGCAGAAGACGGCATACGAGATNNNNNNNNGTGACTGGAGTTCAGACGTGTGCTCTTCCGATCTGAC. The second PCR was conducted under the same protocol described above, except only eight cycles were performed. Libraries were purified with QIAquick 96 PCR Purification Kit (QIAGEN), quantified using Quant-iT PicoGreen dsDNA Assay Kit (Thermo Fisher Scientific), pooled in equimolar concentrations, further purified with AMPure XP (Beckman Coulter) and sequenced with 300 bp × 2 paired end-reads on an Illumina NextSeq1000 using the NextSeq 1000/2000 reagent kit (Illumina).

### Data processing and bioinformatic analysis

Raw Illumina paired-end reads were processed using QIIME2 (version 2024.5). To remove the index primer sequences, 23 bases were trimmed from the 3′ end of both forward and reverse reads. Reads with high similarity to human genome sequences (GRCh38) or PhiX contamination sequences were excluded. The resulting paired-end reads were trimmed, denoised, merged and chimeric sequences were removed to generate amplicon sequence variants (ASVs) using the DADA2 plugin (Caporaso et al. 2010, Mohsen et al. 2019). A custom *Faecalibacterium rpoA* reference library was curated, consisting of 106 *rpoA* sequences from 12 species, with at least one representative sequence per species (Table S2). Taxonomic assignment was performed by clustering ASVs against the custom curated *rpoA* reference library at 98% identity. ASVs with similarity ≥ 98% were assigned to *Faecalibacterium* spp. labelled Group 1–12, those with ≥95% to <98% similarity were classified as “Other *Faecalibacterium*”, and those with <95% as “Others”. To ensure data quality, only ASVs with ≥ 250 bp were retained for downstream analysis. Additionally, 16S rRNA gene sequencing data from the previous study (Park et al. 2021) were used for genus-level analysis of *Faecalibacterium*.

### Statistical analysis

To explore the potential host determinants of *Faecalibacterium* composition, analysis was conducted using both genus-level relative abundance (Park et al. 2021) and specific species exhibiting major-to-intermediate relative abundance. Minor species with median relative abundance under 1% or prevalence under 10% (Groups 2, 5, 7, 8, 9, 10, and 12) were not reported this study, since they were only occasionally detected with at very low levels.

For human metadata analysis, the Mann-Whitney U test was used to compare relative abundance of *Faecalibacterium* spp. between two variables, the Kruskal-Wallis test with Bonferroni correction was applied when comparing three or more variables. Statistical analyses were performed using IBM SPSS Statistics for Windows (version 26). A *P*-value of <0.05 was considered statistically significant. *p*^a^ represents Bonferroni-adjusted *P*-values.

To visualize the relationship between dietary intake profiles and the species-level composition of *Faecalibacterium*, Principal Coordinates Analysis (PCoA) was conducted using Bray–Curtis dissimilarity with the “vegan” package in R (v4.3.2) (Oksanen et al. 2025). Clustering was performed with Partitioning Around Medoids (PAM) to identify dietary patterns among participants. The optimal number of clusters was determined by evaluating average silhouette width across a range of k values using the “cluster” package (Maechler et al. 1999). The envfit function was applied to fit vectors onto the ordination space, and PERMANOVA function was conducted with the adonis function.

Microbial associations with dietary and nutrient intake were assessed using the Microbiome Multivariate Association with Linear Models (MaAsLin2) package (Mallick et al. 2021), a multivariable linear framework. Associations were tested using the default linear model, and multiple testing was controlled with Benjamini–Hochberg (BH) False Discovery Rate (FDR) correction. In this study, associations with *P* < 0.05 is considered statistically significant. FDR *q*-values < 0.1 were regarded as high confidence, <0.25 for moderate confidence (MaAsLin2 default) and <0.3 as exploratory (Segata et al. 2011, Mallick et al. 2021). Given the high dimensionality of microbiome data and the complex physiological interactions between host and diet, applying a more permissive FDR threshold was considered appropriate for this exploratory study.

## Results

### Efficiency and specificity of the designed *Faecalibacterium*-*rpoA* primers determined by qPCR

**Figure 2:**
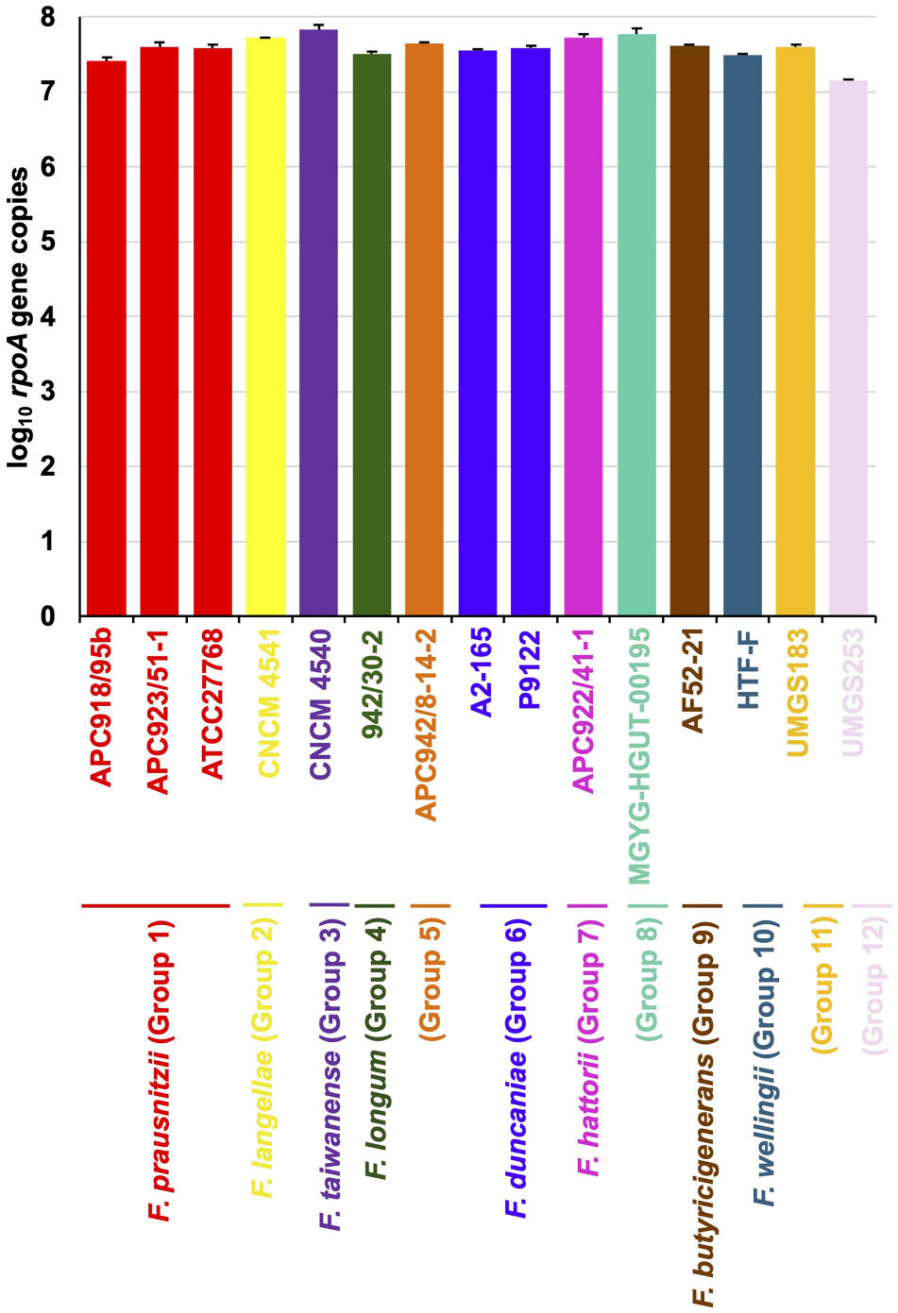
Validation of the designed *Faecalibacterium rpoA*-specific primer set using qPCR. qPCR assays were performed to evaluate the efficiency and specificity of the designed primers. Primer efficiency was determined by qPCR using 15 synthetic *rpoA* DNA samples derived from 12 different *Faecalibacterium* species, each at a concentration of 100 pg (= 7.43 log_10_  *rpoA* gene copies). Bars and error bars represent means and standard deviations, respectively. Bars with the same color indicate an assignment into the same species. All reactions were run in triplicate on the same plate.

qPCR using combination of *Faecalibacterium*-rpoA primer pairs and synthetic *rpoA* DNA from 15 strains in 12 species revealed that all synthetic DNA samples reached similar threshold values to the expected concentration of 7.43 log_10_  *rpoA* gene copies (Fig. 2), indicating consistent amplification across species. This result demonstrates consistent amplification efficiency across the full range of species targeted. Next, primer specificity was investigated using 10 ng of DNA extracted from pure cultures of human commensal bacteria and *Faecalibacterium* strains. No amplification was detected from 11 different non-targeted commensal bacteria, while only the targeted *Faecalibacterium* strains yielded amplification. All four *Faecalibacterium* cultures were quantified at the similar level, *F. langellae* CNCM I-4541^T^ (mean ± SD of log_10_  *rpoA* gene copies, 6.69 ± 0.05), *F. taiwanense* JCM 39529^T^ (6.52 ± 0.11), *F. longum* JCM 39211^T^ (6.52 ± 0.35), and *F. duncaniae* JCM 31915^T^ (6.27 ± 0.51) (Figure S1). Together, these results demonstrate that the designed *Faecalibacterium*-*rpoA* primer pair exhibits both high efficiency and specificity, suggesting that it is suitable for analyzing *Faecalibacterium* composition in human fecal samples.

### Species level composition of *Faecalibacterium* spp. in the Japanese adult population revealed by *rpoA* sequencing

**Figure 3:**
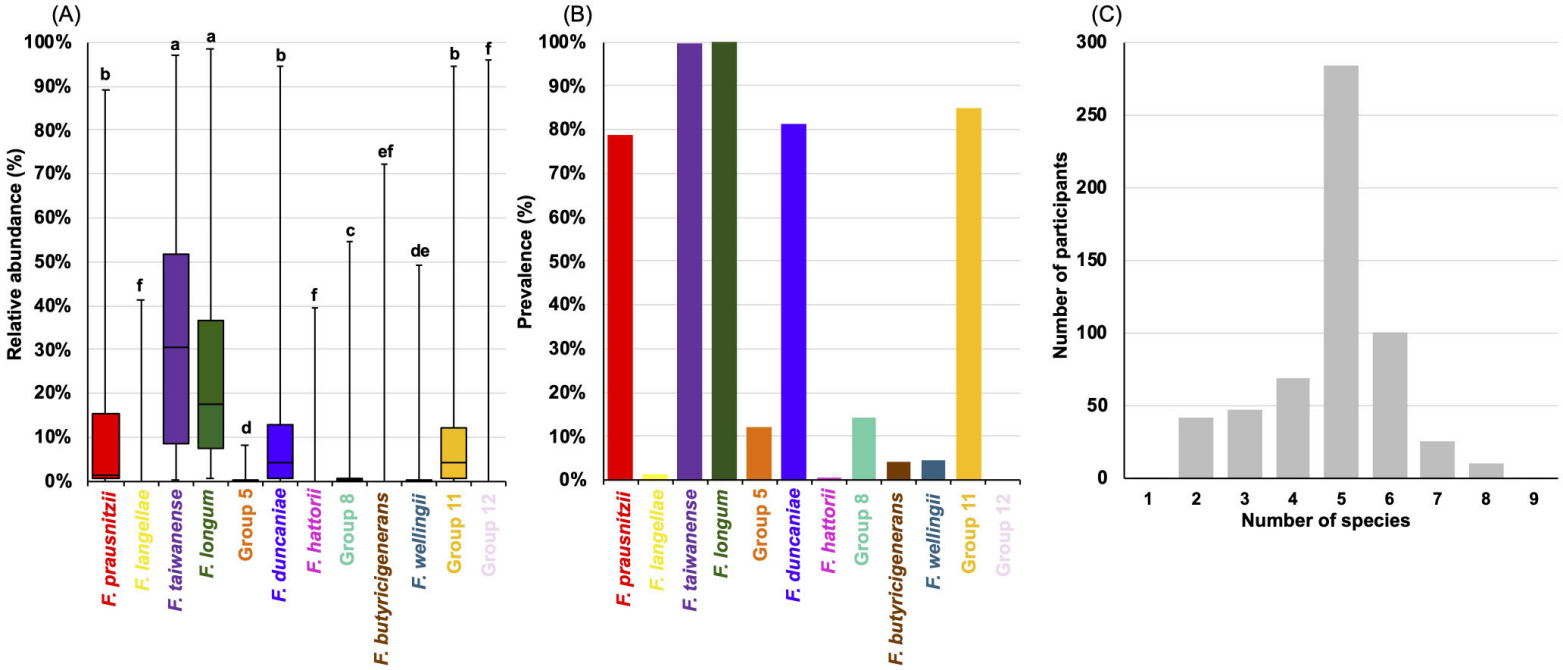
Distribution of *Faecalibacterium* spp. among 580 adult Japanese participants based on *rpoA* sequencing. Box plot showing the relative abundance of *Faecalibacterium* species of human origin within the IQR (A). Bar graph showing the prevalence (%) of each *Faecalibacterium* species among participants (B). Bar graph representing the number of *Faecalibacterium* species coexisting within each individual (C). Different letters in (A) indicate significant differences (*P*^a^ < 0.05, Bonferroni-adjusted *P*-values).

The newly validated *rpoA* sequencing assay was applied to 580 adult Japanese subjects to study species level composition within the *Faecalibacterium* genus. Our study revealed that the *Faecalibacterium* community is largely dominated by two species, i.e. *F. taiwanense* [median ± interquartile range (IQR, Q1–Q3), 30.64% ± 8.58%–51.69%] and *F. longum* (17.40% ± 7.38%–36.78%). Then followed by an intermediate relative abundance consisting of *F. duncaniae* (4.43% ± 0.72%–13.04%), Group 11 (4.39% ± 0.78%–12.13%), and *F. prausnitzii* (1.48% ± 0.60%–15.26%). The median relative abundances of remaining seven groups (Groups 2, 5, 7, 8, 9, 10, and 12) were below 1% (Fig. 3A), suggesting that these represent the minor *Faecalibacterium* community in the human gut. To identify other potential *Faecalibacterium* species that have not yet been characterized, we categorized ASVs with ≥ 95% to < 98% sequence similarity as “Other *Faecalibacterium*”. This group exhibited a very low relative abundance (median ± Q1–Q3: 0.0% ± 0.0%–0.0%) and a prevalence of 4.7%, suggesting that uncharacterized *Faecalibacterium* species are rare within the subjects tested.

Analysis on prevalence (defined as ≥ 0.5% relative abundance) confirmed the widespread detection of major species, with *F. longum* (580/580, 100.0%) and *F. taiwanense* (579/580, 99.8%) in nearly all participants, followed by Group 11 (493/580, 85.0%), *F. duncaniae* (471/580, 81.2%) and *F. prausnitzii* (456/580, 78.6%). The remaining species had prevalence below 15% (Fig. 3B). On average, participants harbored 5 ± 1.28 (median ± SD) *Faecalibacterium* species, with a maximum of nine species (n = 1) and a minimum of one species (n = 1) detected per subject (Fig. 3C).

### Associations between *Faecalibacterium* species composition and Human metadata

**Figure 4:**
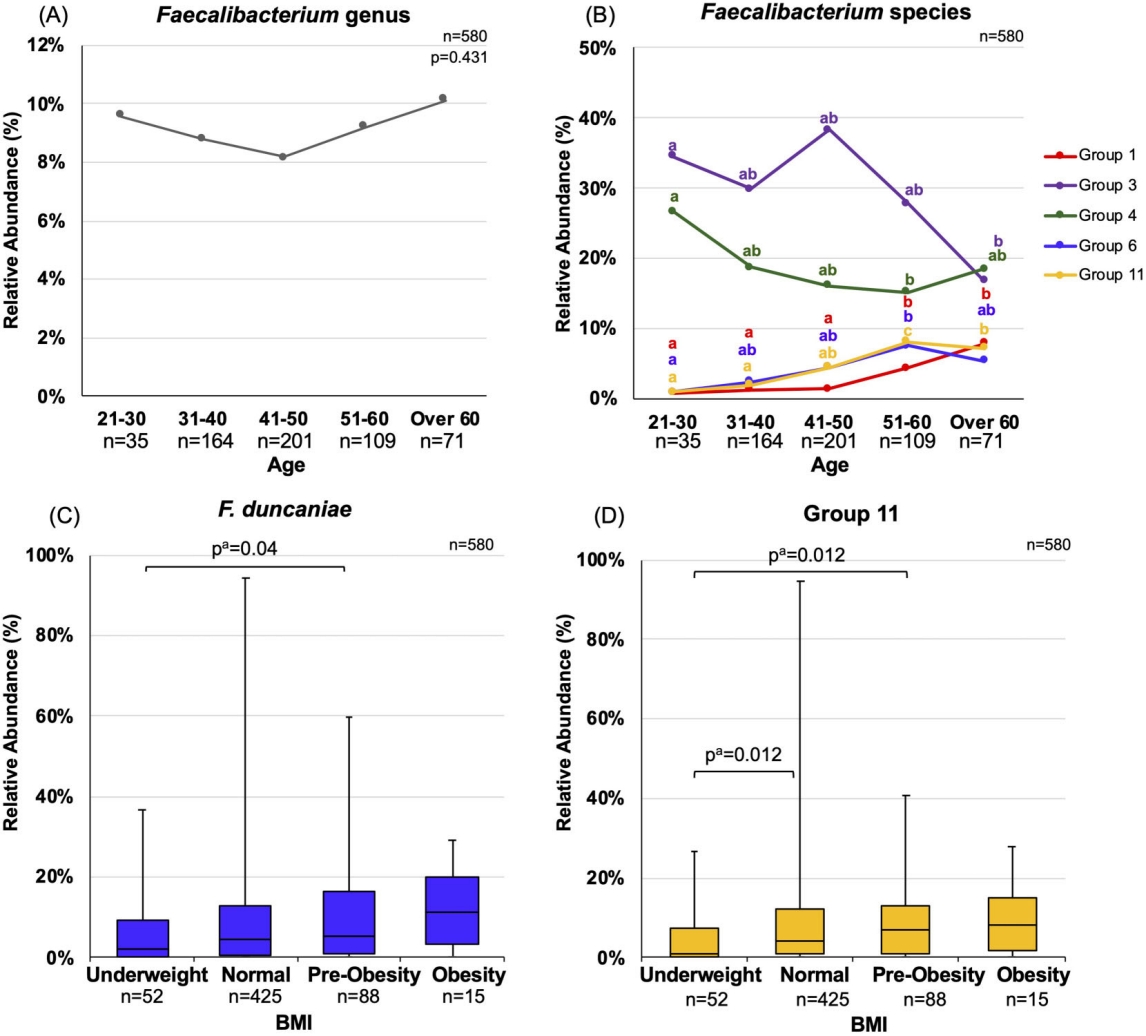

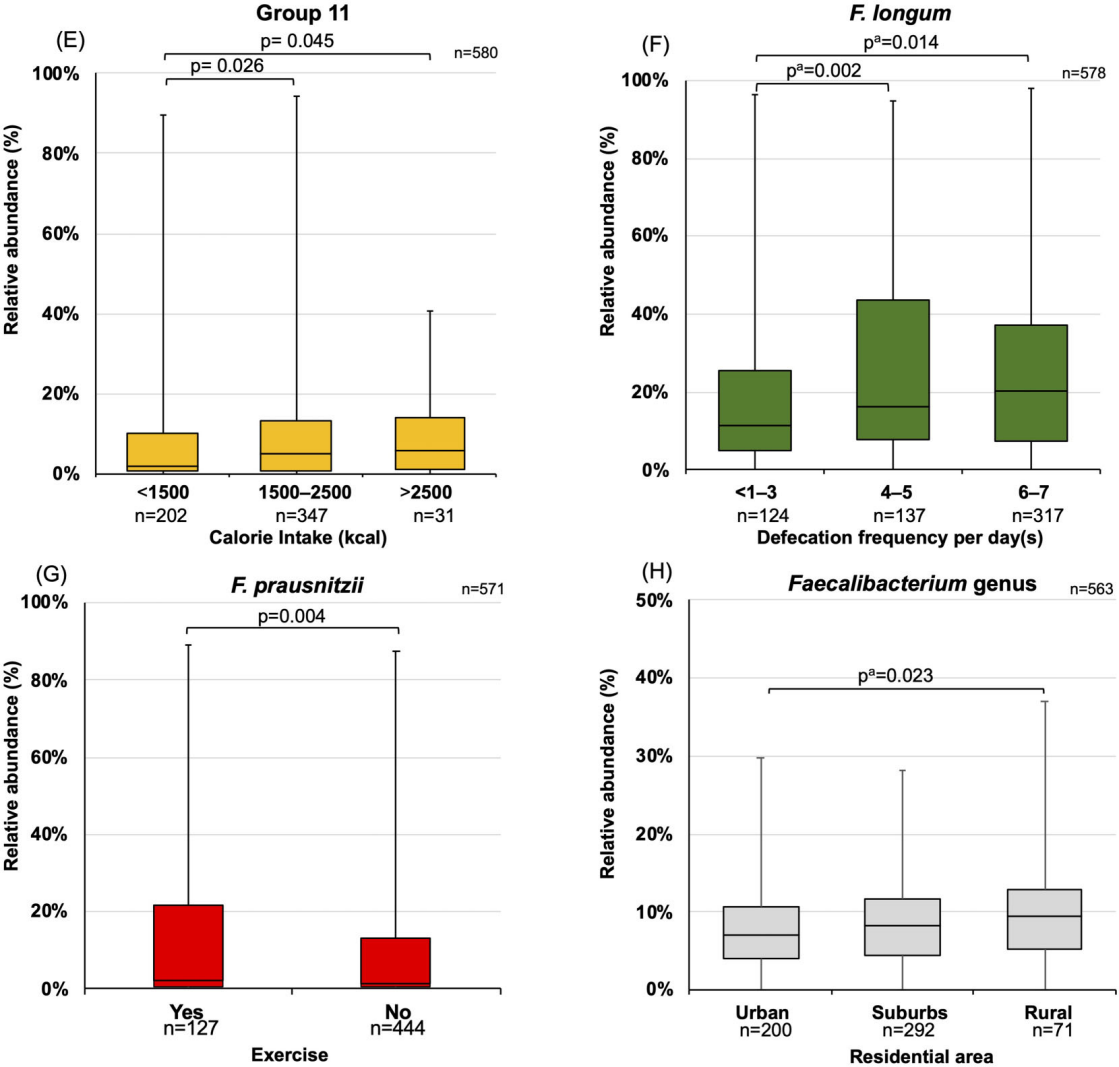
Relative abundance of *Faecalibacterium* genus and species across human metadata factors. Line graphs showing the median relative abundance of *Faecalibacterium* genus, as determined by 16S rRNA gene sequencing,(A) and of individual species, as determined by *rpoA* sequencing, (B) by age. Box plots showing the IQR of relative abundance for *F. duncaniae* (C) and Group 11 (D) across BMI categories; Group 11 across different levels of calorie intake (E); *F. longum* grouped by defecation frequency (F); and *F. prausnitzii* stratified by exercise habits (G). (H) Box plot showing *Faecalibacterium* genus abundance by residential area. Mann–Whitney U tests were used for comparisons between two groups, and Kruskal–Wallis tests for comparisons among more than three groups. Participants with missing data were excluded. *P* < 0.05 was considered significant. (A-B) Different letters indicate significant differences (*P*^a^ < 0.05, Bonferroni-adjusted *P*-values).

For age, participants were grouped in 10-year intervals, with those aged 60 and above categorized into a single group. In our study, relative abundance of *Faecalibacterium* genus, as determined by 16S rRNA gene sequencing in a previous study (Park et al. 2021), remained consistent throughout all age groups (*P* = 0.431) (Fig. 4A), although differences were observed at the species level (Fig. 4B). *Faecalibacterium prausnitzii*, Group 11 and *F. duncaniae* showed an increasing trend with age. The relative abundance of *F. prausnitzii* increased with age, with participants aged 51–60 and those over 60 showing significantly higher levels than those aged 21–30, 31–40, and 41–50. Group 11 showed a similar increasing trend in participants aged 51–60 and over 60, with significantly higher relative abundance than those aged 21–30 and 31–40. Moreover, 51–60 age group had significantly higher levels than both the 41–50 and over-60 groups for Group 11. For *F. duncaniae*, participants aged 51–60 had higher relative abundance than those aged 21–30. In contrast, the relative abundance of *F. longum* and *F. taiwanense* declined with age. The levels of *F. longum* were lower in the 51–60 age group, and *F. taiwanense* levels were reduced in participants over 60, compared to those aged 21–30 (Fig. 4B).

Next, the BMI of 580 participants were categorized based on World Health Organization (WHO) standards. Obese participants (BMI ≥ 30) were a minority (<3%) in this study cohort. Our study found that underweight participants (BMI < 18.5) have significantly lower relative abundance of specific *Faecalibacterium* species in comparison to participants with higher BMI, i.e. normal BMI (18.5 < BMI < 24.9; Group 11, *P*^a^ = 0.012) and pre-obese participants (25.0 < BMI < 29.9; *F. duncaniae, P*^a^ = 0.04; Group 11, *P*^a^ = 0.012) (Fig. 4C and D). Further analysis was also conducted based on estimated daily caloric intake. The daily caloric intake was separated into three different groups respectively: < 1500 kcal, 1500–2500 kcal, and > 2500 kcal. Higher relative abundance of Group 11 was observed in both higher caloric intake groups of 1500–2500 kcal and over 2500 kcal (*P* = 0.026, *P* = 0.045) than the lower caloric intake group (<1500 kcal) (Fig. 4E).

Regarding lifestyle habits, participants with higher frequency of defecation on a day-per-week basis (4–5 days/week and 6–7 days/week) was observed to have a higher relative abundance of *F. longum* (*P*^a^ = 0.002 and *P*^a^ = 0.014, respectively) than the lower defecation frequency group (<1–3 days/week) (Fig. 4F). Participants who reported to have a regular habit of high-impact exercise (at least twice a week, over 30 min a day for over a year) had a higher proportion of *F. prausnitzii* (*P* = 0.004) in comparison to those without (Fig. 4G).

Residential area showed no significant difference on the species level; however, in comparison to the urban region, participants from rural area (*P*^a^ = 0.023) had a higher relative abundance of *Faecalibacterium* genus (Fig. 4H).

### Exploration of potential biomarker: food and nutritional intake

Using dietary data from BDHQ, we examined associations between *Faecalibacterium* species composition and dietary intake including food categories (Table S1), macronutrients, and micronutrients.

**Figure 5:**
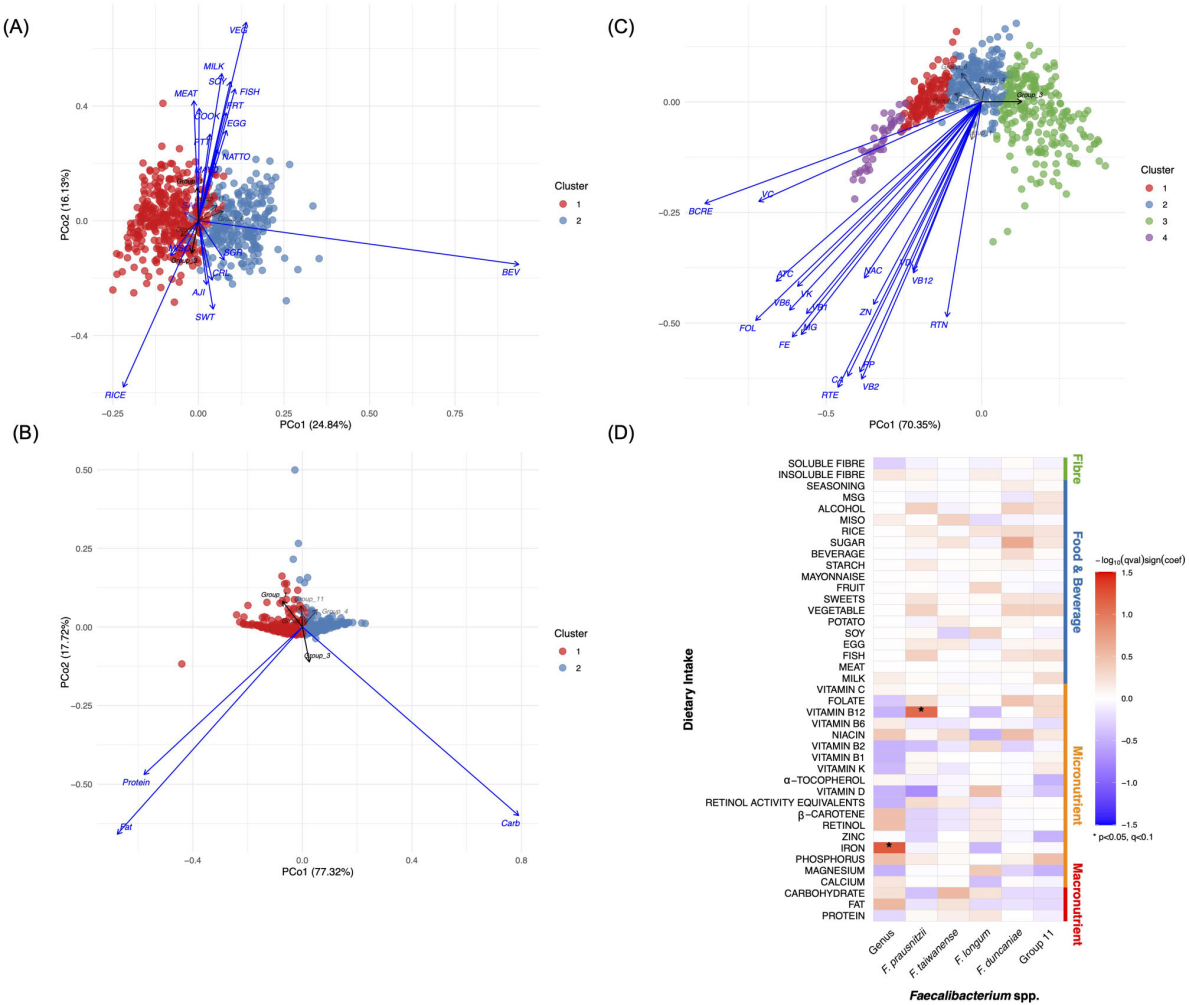
Distribution of *Faecalibacterium* spp. across dietary intake profiles. PCoA based on Bray–Curtis dissimilarity visualizes relationships between dietary intake profiles and *Faecalibacterium* species composition: (A) food categories, (B) macronutrient, and (C) micronutrient intake. Samples were clustered using PAM to identify dietary patterns among participants. Dietary intake within each cluster is shown in Figure S2. The *envfit* function was used to fit vectors onto the ordination, and PERMANOVA was applied to assess significance. Percent variance explained by PCo1 and PCo2 is indicated on the axes. Vectors represent the direction and strength of associations between dietary variables and *Faecalibacterium* species. Significant vectors (*P* < 0.05) are shown in black; nonsignificant vectors are shown with reduced opacity. (D) Heatmap showing correlations between the relative abundance of major *Faecalibacterium* species and dietary intake variables (macronutrients, micronutrients, food/beverage groups, and fiber) analyzed by MaAsLin2, adjusted for BMI, age, and sex. “*P* < 0.05, *q* < 0.1″ according to the Benjamini–Hochberg (BH) false discovery rate (FDR). Abbreviation: CA, calcium; MG, magnesium; PP, phosphate, FE, iron; RTN, retinol; BCRE, β-carotene equivalent; RTE, retinol equivalent; VD, vitamin D; ATC, α-tocopherol; VK, vitamin K; VB1, vitamin B_1_; VB2, vitamin B_2_; NAC, niacin; VB6, vitamin B_6_; VB12, vitamin B_12_; FOL, folate; VC, vitamin C. Food categories are described in Table S1.

First, we studied the correlation between food categories and relative abundance of *Faecalibacterium* species (Fig. 5A). *Faecalibacterium prausnitzii* showed significant positive associations with protein-rich food categories such as MILK, MEAT, EGG, FISH, and SOY. In contrast, *F. taiwanense* was positively associated to carbohydrate-based food categories such as RICE, CRL (which includes bread and noodles), SWT (which consists of several sweets), and SGR (sugar intake) (Fig. 5A). To further characterize the nutrient profiles underlying these clusters, we examined dietary intake distribution across PAM-defined clusters (Figure S2A). Among food categories, beverage (BEV) intake varied significantly across clusters (FDR-adjusted *P* < 0.05; Table S3). MaAsLin2 identified a positive association between sugar intake and *F. duncaniae* (coef = 0.29, *P* = 0.00, *q* = 0.22; Table S4).

Regarding macronutrients, *F. prausnitzii* was negatively associated with carbohydrate intake (Fig. 5B). All macronutrients differed significantly across clusters (FDR-adjusted *P* < 0.05; Figure S2B, Table S3). MaAsLin2 results indicated an exploratory positive correlation between *F. taiwanense* and carbohydrate intake (coef = 0.22, *P* = 0.04, *q* = 0.29, Table S4). For micronutrients, *F. taiwanense* responded along the multivariate nutrient gradient (Fig. 5C), with all micronutrients differing across clusters (FDR-adjusted *P* < 0.05; Figure S2C, Table S3). MaAsLin2 analysis showed that *F. prausnitzii* was positively correlated (coef = 1.34, *P* = 0.00, *q* = 0.08, Fig. 5D) with Vitamin B_12_ and negatively correlated with Vitamin D (coef = −1.2, *P* = 0.00, *q* = 0.19, Table S4).

At the genus level, iron (FE) intake was positively associated with *Faecalibacterium* genus abundance (coef = 0.65, *P* = 0.003, *q* = 0.06; Fig 5D). No significant associations were observed between the genus for food or macronutrient intake. Dietary fiber, both soluble and insoluble, showed no significant correlation at either genus or species level in this study (Fig. 5D).

## Discussion

Our study employed the *rpoA* gene to resolve species-level diversity within *Faecalibacterium*, overcoming non-exploratory limitations of qPCR and insufficient discriminatory power of 16S rRNA sequencing. Notably, our approach uncovered species-specific host-associated traits that were obscured at the genus level. While 16S rRNA gene analysis showed no significant associations (except geographical region), species-level analysis detected specific associations with age, BMI, calorie intake and more. This suggests that genus-level abundance remains relatively stable across demographic groups, concealing species-level compositional shifts.

The developed *rpoA*-based sequencing revealed that *F. longum* and *F. taiwanense* emerged as the most predominant and prevalent species within the 12 human-origin species in Japanese adults. These results align with recent findings by Hirasaki et al. in a separate Japanese cohort (Hirasaki et al. 2025) and Mclellan et al. in a French adult cohort, (Mclellan et al. 2025), both of whom identified *F. taiwanense* and *F. longum* as the dominant taxa. Similarly, the metagenomic study by De Fillippis et al. also reported a high global prevalence of *F. taiwanense* (78%; described as “Clade A” in the article) and *F. longum* (73%; Clade D), though abundance of these species was not discussed (De Filippis et al. 2020). We therefore conclude that these two species consistently represent primary constituents of the human *Faecalibacterium* population.

Prior to the reclassification of *Faecalibacterium, in vitro* studies have included strains originally assigned to *F. prausnitzii* that were later reclassified into distinct species. Among these, strains now classified as *F. duncaniae* exhibited both the highest butyrate production (Martín et al. 2017) and the most potent MAM-mediated anti-inflammatory property (Auger et al. 2022), and strains currently classified as *F. longum* and *F. taiwanense* displayed intermediate immunomodulatory properties, characterized by moderate butyrate production and MAM-mediated anti-inflammatory properties. More recently, the administration of *F. longum* in DSS-induced mice and high-fat-diet-induced obese mice demonstrated protective effects, including the reduction of pro-inflammatory cytokines (IL-1β, IL-6, and TNF-α) and the strengthening of intestinal barrier (Li et al. 2025, Wang et al. 2025). Despite these findings, species-level characterization remains limited, understanding the need for comprehensive studies of the functional roles of individual species in host health.

Following the two major species, we observed *F. duncaniae*, a yet-to-be classified species termed “Group 11″, and *F. prausnitzii* as the next most abundant taxa. Prior to the recent reclassification, *F. prausnitzii* was widely regarded as one of the most predominant species in healthy gut microbiota and a definitive marker of gut health (Sokol et al. 2008, Lopez-Siles et al. 2017). However, our findings supported by recent reports (Hirasaki et al. 2025, Mclellan et al. 2025), suggests that *F. prausnitzii* is not the dominant constituent of *Faecalibacterium* composition. In our cohort, the median relative abundance of *F. prausnitzii* was only 1.48% (IQR: 0.60%–15.26%), with detection in 78.6% of participants. This indicates that more abundant and prevalent species, such as *F. longum, F. taiwanense, F. duncaniae*, and Group 11, may play equally or more important roles in host health.

Notably, this is the first study to characterize in detail the prevalence and relative abundance of the yet-to-be-classified Group 11 within a large human cohort, thereby establishing it as one of the major *Faecalibacterium* species. At the time of writing (May 2025), Group 11 has been detected exclusively through metagenome-assembled genomes (MAGs) in public databases and remains uncultured. We hypothesize that this may be due to fastidious physiological traits, such as extreme oxygen sensitivity or specific nutritional requirements of Group 11, which have thus far prevented successful isolation. To identify other potentially major human *Faecalibacterium* species yet to be characterized, we defined a category termed “Other *Faecalibacterium*”. This group showed negligible prevalence and relative abundance, suggesting that Groups 1–12 likely encompass the majority of *Faecalibacterium* species present in the human gut.

Our study then characterized the associations between *Faecalibacterium* species composition and human metadata. The gut microbiota is known to shift throughout the lifespan under the influence of age, diet, and environmental factors. While no significant age-related changes were observed at the genus level, species-level variation was evident. Older participants exhibited higher relative abundance of *F. prausnitzii*, Group 11 and *F. duncaniae*, while *F. longum* and *F. taiwanense* declined with age. These species-specific shifts align partially with Hirasaki, et al., who reported the increased *F. duncaniae* in middle-aged adults (40–50 s).

Previous studies in elderly populations using 16S rRNA gene sequencing have associated decrease in the *Faecalibacterium* genus with age-related conditions such as frailty (Xu et al. 2021), heart failure (Zhang et al. 2025) and sarcopenia (Liu et al. 2025). Lv et al. also reported a positive association between *F. prausnitzii* and muscle health in older menopausal women (Lv et al. 2021), while the work was conducted before the reclassification of *F. prausnitzii* (Sakamoto et al. 2022). We hypothesize that there are deeper nuances within the genus, whereby specific *Faecalibacterium* species may contribute to healthy aging through butyrate production and MAMs. Longitudinal studies are needed to establish whether these compositional shifts drive age-related health outcomes or represent consequences of the aging process. Furthermore, comparative studies between diseased cohorts and healthy, age-matched controls are needed to validate the clinical relevance of these shifts and to identify specific protective microbes for promoting healthy aging.

Geographical region also influenced *Faecalibacterium* composition. Participants in rural area showed higher genus-level abundance than urban populations, though no species-level shifts were detected. Geographic differences in gut microbiota are often attributed to dietary patterns. Urban populations are often characterized by western dietary habits, represented by high consumption of animal protein and simple sugar with reduced intake of dietary fiber (Conteh and Huang 2020). Contrastingly, rural communities typically maintain fiber-rich, plant-based diets (*Oliver* et al.). Similar to our findings, Park et al. reported that *Faecalibacterium* was more frequently detected in a “longevity village” than in urbanized communities of South Korea (Park et al. 2015). Conversely, another study revealed that Hadza gatherers show depleted *Faecalibacterium* in comparison to an urbanized Italian population (Schnorr et al. 2014), while an urbanized Tibetan population showed higher relative abundance of *Faecalibacterium* than traditional herdsman (Li et al. 2018). These contradicting findings underscore the complex interplay between diet, lifestyle, and gut microbial composition, highlighting varying patterns of microbiota adaptation across populations and levels of urbanization.

Defecation frequency was linked to *F. longum*, which was reduced in participants with lower defecation frequency. Short chain fatty acids (SCFAs) stimulate peristalsis via serotonin signaling in the enteric nervous system (Soret et al. 2010, Vincent et al. 2018), and constipation has been associated with depletion in SCFA producers (Johnson-Martínez et al. 2024), notably butyrate producers such as *Faecalibacterium* (Zhuang et al. 2019). Ito et al. also reported alleviation of constipation coincided with increased relative abundance of *Faecalibacterium* (Ito et al. 2025). Consequently, alterations in the gut microbiota, including the depletion in *F. longum* may contribute to a loss of protective functions in populations with lower defecation frequency. These shifts potentially play a role in the progression of pro-inflammatory intestinal environment in individuals experiencing constipated.

Moreover, nutritional status also correlated with *Faecalibacterium* composition. We observed decreased relative abundance of *F. duncaniae* and Group 11 in participants with underweight BMI and/or lower caloric intake. *F. duncaniae* relative abundance was lower in underweight subjects in comparison to pre-obesity group. Similarly, Group 11 showed lower relative abundance in underweight group compared to normal and pre-obese BMI, as well as in participants with lower caloric intake (<1500 kcal) compared to those with higher calorie intake (1500–2500 kcal and >2500 kcal). Yuan et al. reported a similar finding, showing that underweight anorexia nervosa patients (BMI: 14.92  ±  2.54  kg/m^2^) had significantly lower relative abundance of *Faecalibacterium* spp. compared to healthy controls with normal BMI range (20.89  ±  2.14 kg/m^2^) (Yuan et al. 2022). We hypothesize that caloric intake below the host’s metabolic requirements may selectively disadvantage for *F. duncaniae* and Group 11 by limiting the availability of diet-derived substrates required for microbial proliferation. Additionally, *Faecalibacterium* spp. participate in cross-feeding relationships with other commensal bacteria such as butyrate synthesis from acetate produced by *Bifidobacterium* (Rios-Covian et al. 2015). Therefore, depletion in surrounding acetate-producing commensals driven by limited nutritional availability could also impair growth of *F. duncaniae* and Group 11.

Building on this initial profiling, our study explored the relationship between *Faecalibacterium* and dietary and nutrient intake. PCoA analysis revealed divergence in species distribution across different nutrient profiles. MaAsLin2 analysis revealed a positive association between *F. prausnitzii* and Vitamin B_12_ (cobalamin) intake, consistent with similar associations at the genus level reported by Gurwara et al. (2019). Cobalamin is an essential cofactor for microbial enzymatic reactions (Fischbach and Sonnenburg 2011) and regulates gene expression (Nahvi et al. 2002). The positive correlation between cobalamin intake and *Faecalibacterium* spp. observed in our study may reflect auxotrophy of the organism for this vitamin (Heinken et al. 2014). Together, these species-specific associations with dietary intake suggest metabolic divergence among species, implying distinct ecological niches within the gut ecosystem.

In conclusion, we developed the *rpoA*-based sequencing approach to study the composition of *Faecalibacterium* spp. in the human gut, enabling comprehensive species-level profiling of this genus. Our findings provide a valuable reference for understanding *Faecalibacterium* distribution following recent taxonomic reclassification and reveal human metadata-driven patterns and exploratory associations linking bacterial abundance to dietary and nutritional intake. This study lays the groundwork for future mechanistic and longitudinal studies aimed at elucidating causal relationships. Future application of the developed method to various disease cohorts may help reveal how specific *Faecalibacterium* species are depleted during disease progression. Furthermore, understanding whether compositional shifts are disease-specific or reflect universal patterns of host health could guide the development of next generation probiotics (Martín et al. 2017, Kumari et al. 2021) and targeted biotherapeutics, as well as identifying potential microbial biomarkers for disease diagnostics, as suggested by previous studies (Lopez-Siles et al. 2017).

## Supplementary Material

fiag049_Supplemental_File

## Data Availability

*Faecalibacterium-rpoA* sequencing data has been deposited in DDBJ under accession numbers DRR795574-DRR796153.
