# Supplementary material for: Species level composition of Faecalibacterium spp. in the gut of Japanese adults revealed by rpoA-based sequencing analysis
Source: FEMS Microbiol Ecol. 2026 May 9;102(6):fiag049. doi: 10.1093/femsec/fiag049 (PMC13214989; doi:10.1093/femsec/fiag049)
Supplement: fiag049_Supplemental_File [file fiag049_supplemental_file.pdf]

Supplementary Figure 1. Validation of the designed *Faecalibacterium rpoA*-specific primer set using qPCR. Primer specificity was assessed by qPCR using DNA from 11 commensal gut bacteria and three cultured *Faecalibacterium* strains (10 ng each). Bars and error bars represent means and standard deviations, respectively. Bars with the same color indicate an assignment into the same species. All reactions were run in triplicate on the same plate. ND, not detected.

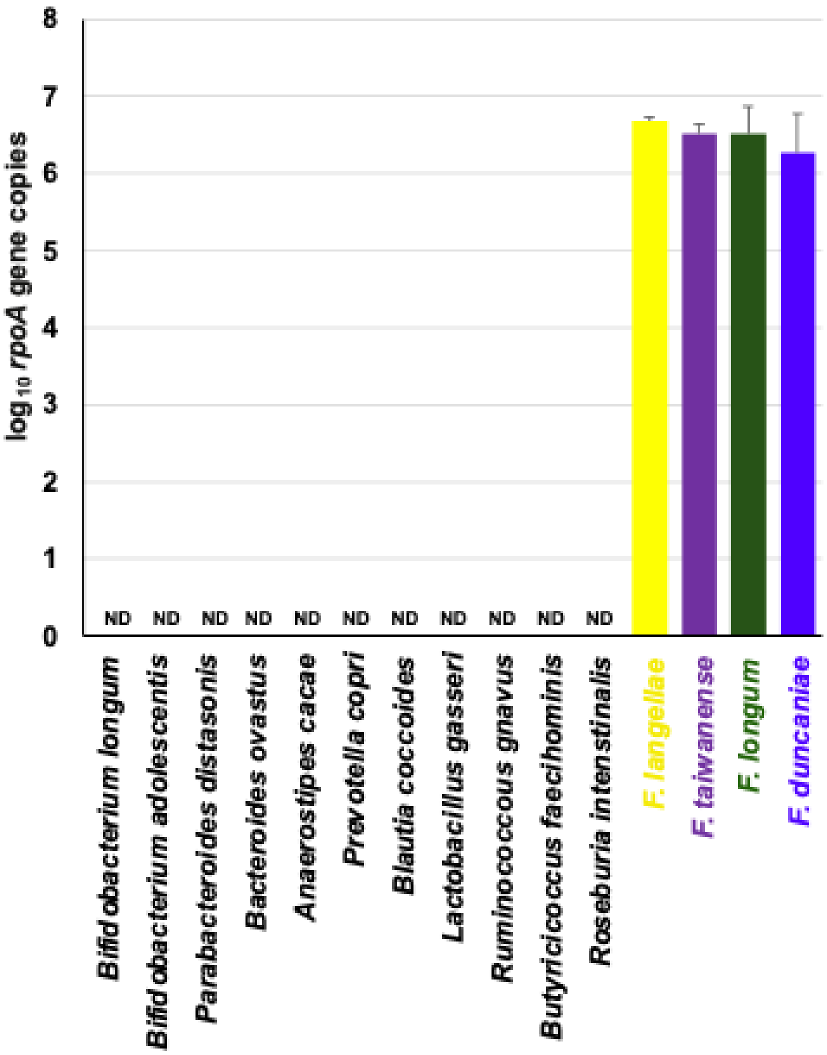

Supplementary Figure 2. Dietary distribution across clusters.
Box plots showing dietary intake per cluster identified in PCoA: (A) food categories, (B) macronutrients, and (C) micronutrients.

(A)

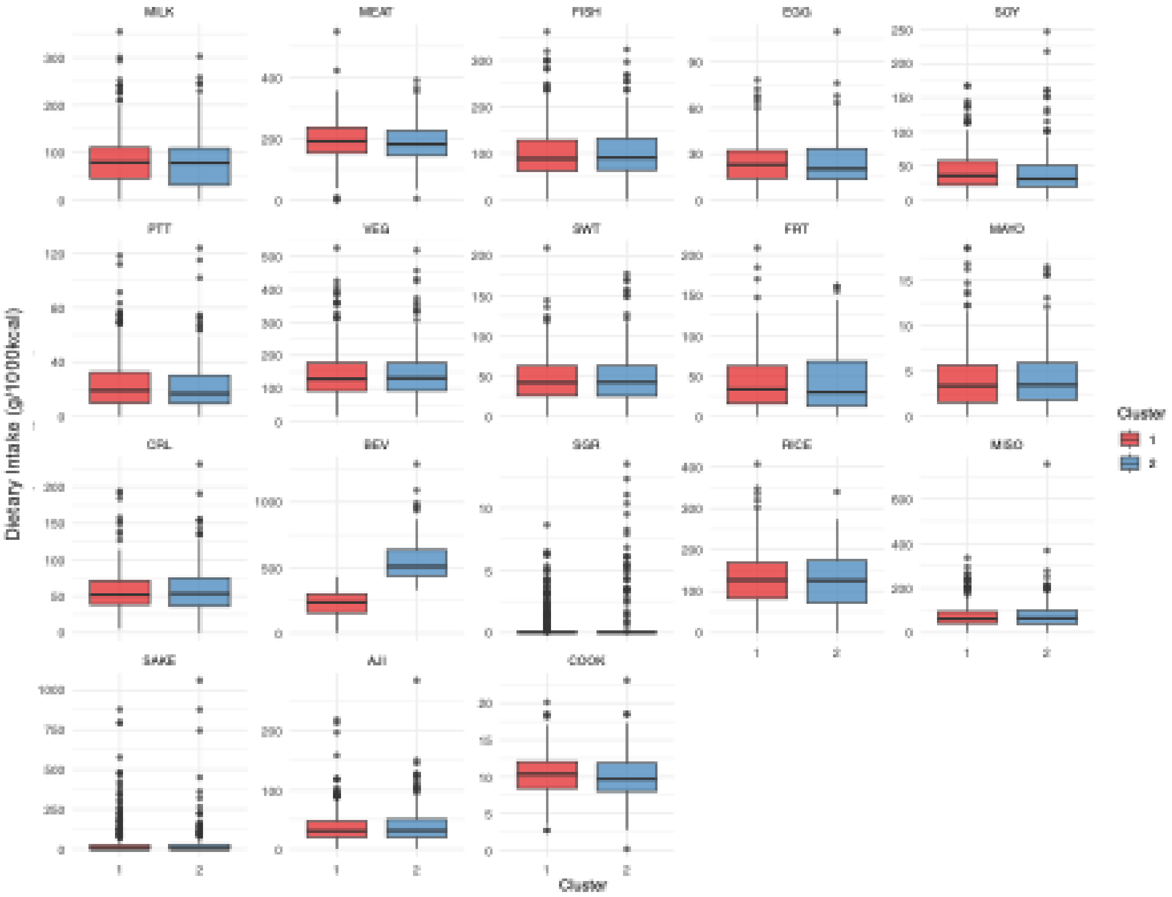

(B)

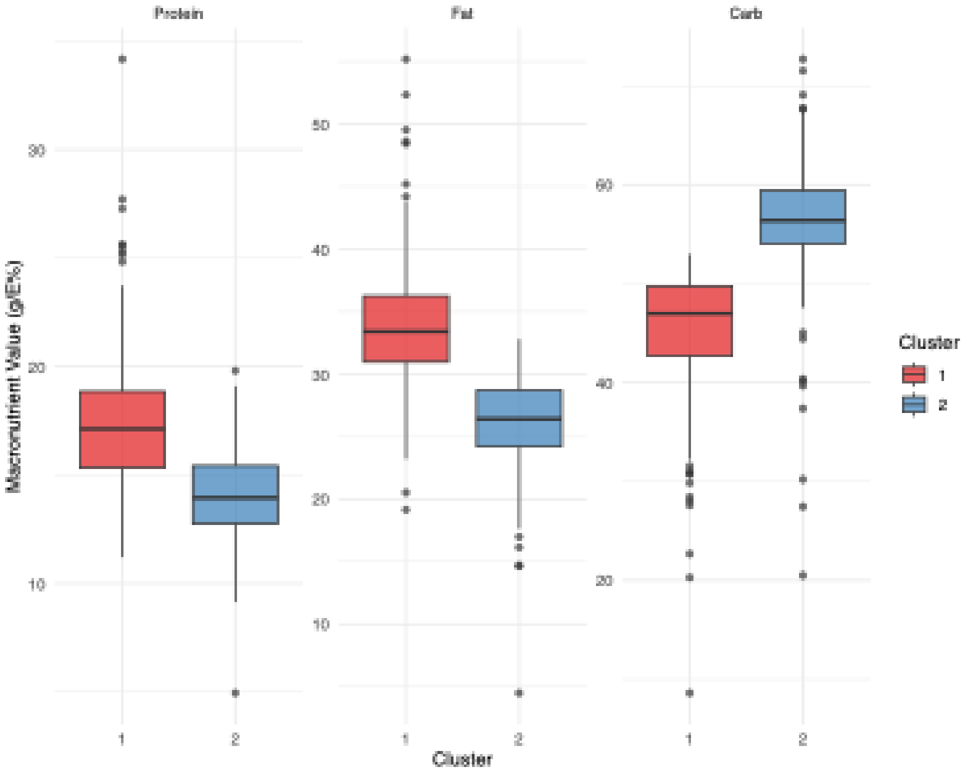
Protein
Fat
Carb
Macronutrient Value (g/E%)
30
20
10
50
40
30
20
10
60
40
20
1
2
1
2
1
2
Cluster
Cluster
1
2

(C)

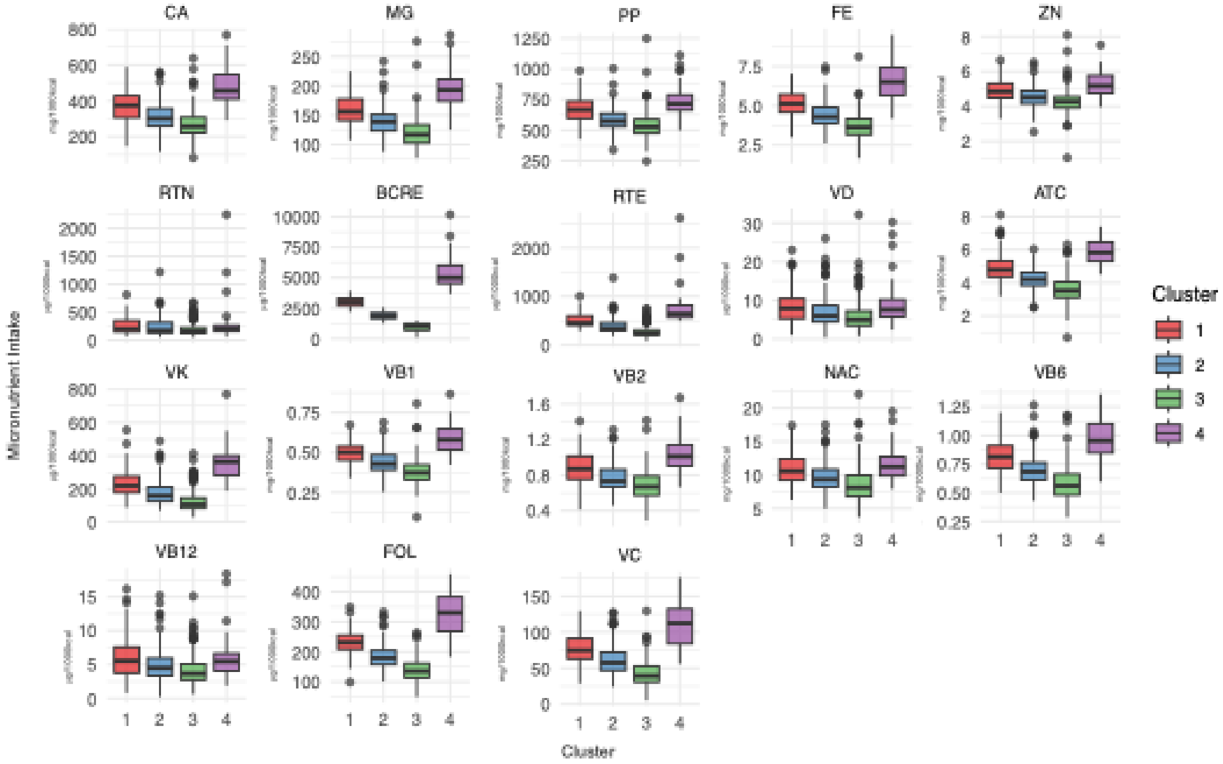

CA
MG
PP
FE
ZN
RTN
BCRE
RTE
VD
ATC
VK
VB1
VB2
NAC
VB6
VB12
FOL
VC
Micronutrient Intake
Cluster
Cluster
1
2
3
4

Table S1. 67 types of food and beverages divided into 18 different categories based on Brief Self-Administered Diet History Questionnaire (BDHQ).

Category		Food Type
**1**	**MILK**	Low-fat milk
		Regular milk
**2**	**MEAT**	Chicken
		Pork/Beef
		Ham
		Grilled meat
		Hamburger steak
		Fried meat
		Stir-fried meat
		Simmered meat
		Liver
**3**	**FISH**	Raw fish
		Grilled fish
		Simmered fish
		Tempura/Fried fish
		Squid, Octopus, Shrimp, Shellfish
		Fish with bones
		Canned tuna
		Dried fish
		Fatty fish
		Lean fish
**4**	**EGG**	Eggs
**5**	**SOY**	Tofu, Fried tofu
		Natto
**6**	**POTATO**	Potatoes
**7**	**VEGETABLE**	Pickles (leafy vegetables)
		Pickles (other)
		Raw (lettuce, cabbage)
		Leafy vegetables
		Cabbage
		Carrot, Pumpkin
		Radish, Turnip
		Root vegetables
		Tomato

		Mushrooms<br>Seaweed
**8**	**SWEETS**	Western-style sweets<br>Japanese sweets<br>Rice crackers<br>Ice cream
**9**	**FRUIT**	Citrus fruits<br>Strawberries<br>Others
**10**	**MAYONNAISE**	Mayonnaise
**11**	**STARCH(CRL)**	Bread<br>Soba<br>Udon<br>Ramen<br>Pasta
**12**	**BEVERAGE**	Green tea<br>Black tea, Oolong tea<br>Coffee<br>Cola<br>100% Juice
**13**	**SUGAR**	Sugar
**14**	**RICE**	Rice
**15**	**MISO**	Miso soup
**16**	**ALCOHOL**	Sake<br>Beer<br>Shochu<br>Whiskey<br>Wine
**17**	**MSG**	Noodle soup seasoning mix<br>Soy sauce
**18**	**SEASONING**	Cooking salt<br>Cooking oil<br>Cooking sugar

Table S2. List of strains used for curating *rpoA* library in present study.

No.	Strain	Assembly accession	Species (Group)
1	APC923/51-1	GCA_003287405.1	
2	2789STDY5834970	GCA_001406255.1	
3	CNCM4644	GCA_002550015.1	
4	BIOML-B5	GCA_009679995.1	
5	BIOML-B6	GCA_009680005.1	
6	BIOML-B11	GCA_009679905.1	
7	BIOML-B12	GCA_009679865.1	
8	BIOML-B10	GCA_009679895.1	
9	BIOML-B9	GCA_009679915.1	
10	BIOML-B13	GCA_009679855.1	
11	BIOML-B14	GCA_009679795.1	
12	BIOML-B7	GCA_009679955.1	
13	BIOML-B8	GCA_009679965.1	
14	BIOML-B15	GCA_009679805.1	
15	BIOML-B16	GCA_009679815.1	
16	BIOML-B17	GCA_009679775.1	
17	BIOML-B4	GCA_009680015.1	***F. prausnitzii***
18	BIOML-B18	GCA_009679755.1	
19	BIOML-B2	GCA_009680055.1	
20	BIOML-B3	GCA_009680075.1	
21	aa_0143	GCA_004167405.1	
22	ATCC27766	GCA_003324115.1	
23	ATCC27768	GCA_003324185.1	
24	AF10-13	GCA_003465525.1	
25	AM36-18BH	GCA_003467805.1	
26	LMAG:56	GCA_008680095.1	
27	M21/2	GCA_000154385.1	
28	MGYG-HGUT-01300	GCA_902373685.1	
29	APC918/95b	GCA_003312465.1	
30	BIOML-B1	GCA_009680085.1	
31	AF29-11BH	GCA_003434015.1	
32	AF31-14AC	GCA_003433905.1	
33	AM37-13AC	GCA_003434135.1	

34	APC924/119	GCA_003287475.1	
35	SL3/3	GCA_000209855.1	
36	CNCM4546	GCA_002549935.1	
37	CNCM4573	GCA_002549945.1	
38	CNCM4541	GCA_002549775.1	***F. langellae***
39	LMAG:10	GCA_008681055.1	
40	KLE1255	GCA_000166035.1	
41	MGYG-HGUT-02272	GCA_902385265.1	***F. taiwanense***
42	CNCM4542	GCA_002549895.2	
43	CNCM4540	GCA_002549755.1	
44	CNCM4544	GCA_002549905.1	
45	CM04-06	CNA0017731	
46	APC942/18-1	GCA_003287505.1	
47	942/30-2	GCA_003293635.1	
48	MGYG-HGUT-02545	GCA_902388275.1	
49	SSTS_Bg7063	GCA_902167805.1	
50	FPSSTS7063_SV_a2_mod	GCA_902167865.1	***F. longum***
51	JG_BgPS064	GCA_902167845.1	
52	L2/6	GCA_000210735.1	
53	AF32-8AC	GCA_003433995.1	
54	APC942/32-1	GCA_003287485.1	
55	MGYG-HGUT-02274	GCA_902385305.1	
56	APC942/8-14-2	GCA_003287415.1	**Group 5**
57	APC923/61-1	GCA_003287495.1	
58	P9239	GCA_902497375.1	
59	P9241	GCA_902497405.1	
60	P9240	GCA_902497335.1	
61	P9313	GCA_902497345.1	
62	MGYG-HGUT-00022	GCA_902362495.1	
63	A2-165	GCA_000162015.1	***F. duncaniae***
64	A2165	GCA_002734145.1	
65	P9094	GCA_902497355.1	
66	P9238	GCA_902497365.1	
67	CNCM4543	GCA_002549855.1	
68	CNCM4574	GCA_002549985.1	

69	P9122	GCA_902497435.1	
70	P9391	GCA_902497395.1	
71	AF36-11AT	GCA_003433865.1	
72	AM39-7BH	GCA_003434165.1	
73	AM42-11AC	GCA_003434125.1	
74	AHMP21-2	GCA_002550045.1	
75	AM33-14AC	GCA_003434175.1	
76	Indica	GCA_002586945.1	
77	P9224	GCA_902497415.1	
78	BIOML-A1	GCA_009680105.1	
79	P9311	GCA_902497385.1	
80	P9123	GCA_902497425.1	
81	P9225	GCA_902497325.1	
82	MGYG-HGUT-00039	GCA_902362505.1	
83	APC922/41-1	GCA_003287455.1	***F. hattorii***
84	CNCM4575	GCA_002549975.1	
85	2789STDY5608869	GCA_001406355.1	
86	AHMP21-1	GCA_002550035.1	**Group 8**
87	MGYG-HGUT-00195	GCA_902364275.1	
88	AF52-21	CNA0017730	
89	HGM13282	GCA_900758465.1	***F. butyricigenerans***
90	SRR7721880-bin.52	GCA_905204885.1	
91	HTF-F	GCA_023347535.1	
92	UMGS184	GCA_900539885.1	***F. wellingii***
93	HTF-128	GCA_037478275.1	
94	UMGS183	GCA_900539945.1	
95	ERR9607189_bin.15	GCA_958351215.1	
96	SRR16280040_bin.16	GCA_959598675.1	**Group 11**
97	ERR1600655_bin.52	GCA_937966645.1	
98	SRR341678-bin.6	GCA_905201955.1	
99	UMGS253	GCA_900540455.1	
100	SRS15956992_bin1	GCA_963557555.1	
101	HGM13272	GCA_900771545.1	**Group 12**
102	SRR2912784-mag-bin.20	GCA_905207885.1	
103	P014L1-10	GCA_037248125.1	

104	MGYG-HGUT-02224	GCA_902476985.1
105	L2_057_000M1	GCA_018374075.1
106	ERR318708_bin.32	GCA_937915165.1

Table S3: Mann-Whitney U, Kruskal-Wallis test of nutrients and dietary intake from different clusters separated by PAM.

Dietary Intake	Test	*p*-value	Adjusted *p*-value
MILK	Mann-Whitney U	0.350	0.710
MEAT	Mann-Whitney U	0.030	0.300
FISH	Mann-Whitney U	0.490	0.740
EGG	Mann-Whitney U	0.450	0.740
SOY	Mann-Whitney U	0.190	0.710
PTT	Mann-Whitney U	0.290	0.710
VEG	Mann-Whitney U	0.620	0.860
SWT	Mann-Whitney U	0.780	0.880
FRT	Mann-Whitney U	0.350	0.710
MAYO	Mann-Whitney U	0.780	0.880
CRL	Mann-Whitney U	0.910	0.910
BEV	Mann-Whitney U	0.000	0.000
SGR	Mann-Whitney U	0.260	0.710
RICE	Mann-Whitney U	0.450	0.740
MISO	Mann-Whitney U	0.710	0.880
SAKE	Mann-Whitney U	0.310	0.710
AJI	Mann-Whitney U	0.900	0.910
COOK	Mann-Whitney U	0.070	0.440

Macronutrient	Test	p-value	Adjusted p-value
Protein	Mann-Whitney U	0.000	0.000
Fat	Mann-Whitney U	0.000	0.000
Carb	Mann-Whitney U	0.000	0.000

Micronutrient	Test	p-value	Adjusted p-value
CA	Kruskal-Wallis	0.000	0.000
MG	Kruskal-Wallis	0.000	0.000
PP	Kruskal-Wallis	0.000	0.000
FE	Kruskal-Wallis	0.000	0.000
ZN	Kruskal-Wallis	0.000	0.000
RTN	Kruskal-Wallis	0.000	0.000
BCRE	Kruskal-Wallis	0.000	0.000
RTE	Kruskal-Wallis	0.000	0.000
VD	Kruskal-Wallis	0.000	0.000
ATC	Kruskal-Wallis	0.000	0.000
VK	Kruskal-Wallis	0.000	0.000
VB1	Kruskal-Wallis	0.000	0.000

VB2	Kruskal-Wallis	0.000	0.000
NAC	Kruskal-Wallis	0.000	0.000
VB6	Kruskal-Wallis	0.000	0.000
VB12	Kruskal-Wallis	0.000	0.000
FOL	Kruskal-Wallis	0.000	0.000
VC	Kruskal-Wallis	0.000	0.000

Table S4: Statistical significance from MaAsLin2 analysis.

Bacteria	Metadata	coefficient	p-value	q-value
Faecalibacterium	Iron	0.65	0.00	0.06
F. prausnitzii	Vitamin B12	1.34	0.00	0.08
F. prausnitzii	Vitamin D	-1.20	0.00	0.19
F. duncaniae	Sugar	0.29	0.00	0.22
F. taiwanense	Carbohydrate	0.22	0.04	0.29
